# Safety and Effects of Football in Skeletal Metastatic Prostate Cancer: a Subgroup Analysis of the FC Prostate Community Randomised Controlled Trial

**DOI:** 10.1186/s40798-021-00318-6

**Published:** 2021-04-20

**Authors:** Eik Dybboe Bjerre, Sarah Weller, Mads Hvid Poulsen, Søren Sørensen Madsen, Rie Dybboe Bjerre, Peter Busch Østergren, Michael Borre, Klaus Brasso, Julie Midtgaard

**Affiliations:** 1 The University Hospitals Centre for Health Research, University of Copenhagen, Rigshospitalet, Department 9701, Blegdamsvej 9, 2100 Copenhagen, Denmark; 2 Prostate Cancer Supportive Care Program, Vancouver Prostate Centre, Vancouver, Canada; 3 Department of Urology and Academy of Geriatric Cancer Research, Odense University Hospital, Odense, Denmark; 4 Department of Urology, Hospital of South West Jutland, Esbjerg, Denmark; 5 Herlev and Gentofte Hospital, University of Copenhagen, Hellerup, Denmark; 6 Department of Urology, Herlev and Gentofte Hospital, Herlev, Denmark; 7 Department of Urology, Aarhus University Hospital, Aarhus, Denmark; 8 Copenhagen Prostate Cancer Center, Department of Urology, University of Copenhagen, Rigshospitalet, Copenhagen, Denmark; 9 Department of Public Health, University of Copenhagen, Copenhagen, Denmark

**Keywords:** Soccer, Cancer survivor, Community, Exercise, Rehabilitation

## Abstract

**Background:**

Skeletal metastatic disease excludes many cancer patients from participating in exercise and physical activity due to safety concerns. Empirical evidence from high-quality trials is warranted to guide clinicians and patients.

**Objective:**

To evaluate the safety and potential benefits of high-impact aerobic exercise in patients with prostate cancer with skeletal metastases.

**Design:**

Exploratory subgroup analysis of a pragmatic, multicentre, parallel randomised controlled trial.

**Setting:**

The trial recruited 214 patients from five hospital urological departments in Denmark.

**Participants:**

Patients with prostate cancer with skeletal metastases (*n* = 41).

**Intervention:**

Six months of football training twice weekly at a local club or usual care. Both groups received brief information on physical activity recommendations at the time of randomisation.

**Main Outcome(s) and Measure(s):**

Safety, defined as falls, fractures and hospital admissions. Effects were evaluated on the primary outcome (prostate cancer-specific quality of life) and secondary outcomes (lean body mass, fat mass, hip and spine bone mineral density, and general physical and mental health).

**Results:**

The original trial comprised 214 participants, 41 of whom had skeletal metastases at enrolment. Of these, 22 were allocated to football and 19 to usual care. The trial retention rate was 95% at 12 weeks and 88% at 6 months. Football participants attended 13 sessions on average at 12 weeks and 23 at 6 months. There were two falls, one in each group after 6 months, and no fractures. There were four unplanned hospital admissions in the study period, all four in the usual care group. Statistically significant between-group difference was observed in the primary outcome change in prostate cancer-specific quality of life at 12 weeks (7.6 points [95% CI 0.5 to 15.0]; *P* = 0.038). No statistical changes were found in the secondary outcomes.

**Conclusion:**

The analysis showed that football training was safe in patients with skeletal metastatic prostate cancer and significantly improved quality of life. Larger analyses and/or trials are warranted to confirm the safety of exercise more broadly in cancer patients with skeletal metastatic disease.

**Trial Registration:**

ClinicalTrials.gov, NCT02430792. Date of registration 30 April 2015

**Supplementary Information:**

The online version contains supplementary material available at 10.1186/s40798-021-00318-6.

## Key Points

- This study aimed to investigate whether high-impact aerobic exercise, such as football, is safe in patients with prostate cancer with skeletal metastases.
- The analysis of this pragmatic randomised controlled trial in patients with prostate cancer with skeletal metastases showed that fewer adverse events occurred in the patients playing football compared to those undergoing usual care. Likewise, prostate cancer-specific quality of life improved for the men in the football group compared to those in the usual care group.
- The results suggest that men with skeletal metastatic prostate cancer should not be excluded from exercise and some can even engage in strenuous, high-impact exercise.

## Background

Survival rates in advanced cancer have increased due to treatment advances, as has the number of people living with adverse treatment side effects, including fatigue, emotional distress and decreased physical functioning [1]. Exercise has been identified as an effective strategy that may ameliorate negative treatment side effects and improve physical function [2]. A majority of men with advanced prostate cancer (PCa) develop bone metastases and may experience pain and subsequent skeletal complications such as pathological fracture or spinal cord compression, functional limitations and reduced quality of life (QoL) [3–5]. Globally, cancer exercise guidelines recommend that individuals with bone metastases should avoid inactivity and perform both aerobic and resistance exercises [6, 7]. Despite this, only two randomised controlled trials (RCTs) have evaluated exercise in PCa patients who all have skeletal metastases [8, 9]. These two studies purposely avoided specific loading to the sites of the skeletal metastases. We conducted what is currently the largest RCT evaluating supervised exercise in men with PCa [10, 11]. In the overall study population, mental health, measured using the 12-Item Short Form Health Survey (SF-12), improved by 3.0 points, although no effect was found in relation to PCa-specific QoL. Our analysis showed that in participants playing football, fat mass decreased, lean body mass remained unchanged and hip bone mineral density showed minor improvement (0.008 g/cm^2^) [10].

As safety is a concern in patients with skeletal metastasis, the objective of this subgroup analysis is to further evaluate the potential safety and effects of unrestricted physical exercise, such as football training, in patients with skeletal metastatic prostate cancer (mPCa).

## Methods

An exploratory subgroup analysis was performed of a pragmatic multicentre RCT conducted at five centres in Denmark between 2015 and 2018 that has been previously described in detail [12]. Participants were randomly allocated 1:1 to either community-based football (FG) 1 h, twice weekly for 6 months, or usual care (UC). Patients eligible for study inclusion were diagnosed with PCa and able to complete questionnaires in Danish. All participants provided informed written consent prior to participation, and the trial was conducted in accordance with the Declaration of Helsinki. Patients were excluded if they had undergone prostatectomy within the last 6 weeks and had a hip or spine *t*-score below 2.5. No formal screening was done for painful skeletal metastases, spinal cord compression or cauda equina syndrome at enrolment. However, possible participants were asked to consider if they or their treating physician had advised them to avoid exercise. This subgroup analysis only included patients with skeletal mPCa at enrolment.

### Intervention

The intervention is described in detail in the protocol [12]. However, in relation to increasing the safety of football in patients with prostate cancer with skeletal metastases (including minimising the risk of injuries), it should be noted here that the intervention consisted of 20 min of warm-up (i.e. running, own bodyweight and partner exercises), 20 min of football skill training and 20 min of regular football match play.

### Outcomes

Safety was evaluated using three outcomes: falls resulting in seeking medical assistance, fractures and hospital admissions. The accuracy of these outcomes reported by patients was verified through a review of hospital records.

Effects were evaluated using PCa-specific QoL assessed using the Functional Assessment of Cancer Therapy-Prostate (FACT-P) questionnaire, mental and physical health assessed with SF-12 and body composition (lean body mass and fat mass) and bone mineral density (hip and spine) assessed using dual-energy X-ray absorptiometry.

An exploratory outcome was disease progression, defined by either doubling of prostate-specific antigen or radiologically verified disease progression based on the review of hospital records.

### Statistical Analyses

All analyses were conducted as described in the protocol, with the primary outcome evaluated after 12 weeks and a study period from baseline to 6 months [12]. Effect outcomes were estimated using analysis of covariance that included the baseline value of the outcome and age as a covariate (adjustment). Changes are presented as marginal mean differences between allocation groups with 95% confidence intervals (CIs). Safety outcomes and progression were summarised using descriptive statistics. Fischer’s exact test was used to compare differences between allocation groups. All analyses were conducted with Stata 15.1 (StataCorp LLC, College Station, TX, USA).

## Results

Between June 2015 and February 2017, 214 participants were randomised [10, 11], 41 of whom (FG (*n* = 22) and UC (*n* = 22)) had skeletal mPCa. Table 1 presents participant characteristics. To our knowledge, no participants had painful skeletal metastases, spinal cord compression or cauda equina syndrome.

**Table 1:** Participant characteristics

	Football (***n*** = 22)	Usual care (***n*** = 19)	Total (***n*** = 41)
Age (years)	68.9 (8.4)	67.3 (7.0)	68.2 (7.7)
Employment status			
Paid work	6 (27%)	4 (21%)	10 (24%)
Retired	16 (73%)	15 (79%)	31 (76%)
Education			
No education	3 (14%)	0 (0%)	3 (7%)
Lower secondary education (9th/10th grade)	1 (5%)	1 (5%)	2 (5%)
Vocational education	6 (27%)	7 (37%)	13 (32%)
Upper secondary education (12th grade)	2 (9%)	2 (11%)	4 (10%)
College or higher	10 (45%)	9 (47%)	19 (46%)
Marital status			
Married or living with partner	17 (77%)	17 (89%)	34 (83%)
Others (single, divorced, widowed)	5 (23%)	2 (11%)	7 (17%)
Time since diagnosis, days	1099 (941)	1152 (953)	1123 (935)
ISUP Gleason grading (score)			
Group 2 (3 + 4)	3 (14%)	2 (11%)	5 (12%)
Group 3 (4 + 3)	2 (9%)	4 (21%)	6 (15%)
Group 4 (8)	4 (18%)	3 (16%)	7 (17%)
Group 5 (9–10)	13 (59%)	8 (42%)	21 (51%)
Not known	0 (0%)	2 (11%)	2 (5%)
Treatment at baseline			
Current			
Anti-androgen monotherapy	1 (5%)	1 (5%)	2 (5%)
Castration (surgical or pharmacological)	21 (95%)	18 (95%)	39 (95%)
Previous or current chemotherapy (docetaxel)	9 (47%)	7 (32%)	16 (39%)
Number of co-morbidities			
Zero	6 (27%)	9 (47%)	15 (37%)
One	4 (18%)	3 (16%)	7 (17%)
Two	6 (27%)	3 (16%)	9 (22%)
Three or more	6 (27%)	4 (21%)	10 (24%)
Bone lesion site			
Pelvis	16 (73%)	9 (47%)	25 (61%)
Femur	2 (9%)	5 (26%)	7 (17%)
Rib/thoracic spine	11 (50%)	10 (53%)	21 (51%)
Lumbar spine	8 (36%)	3 (16%)	11 (27%)
Humerus	0 (0%)	1 (5%)	1 (2%)
All regions	2 (9%)	3 (16%)	5 (12%)
Other sites	0 (0%)	1 (5%)	1 (2%)
Unknown	0 (0%)	3 (16%)	3 (7%)

*ISUP* International Society of Urological Pathology

Data are mean (standard deviation) or *n* (%)

**Fig. 1:**
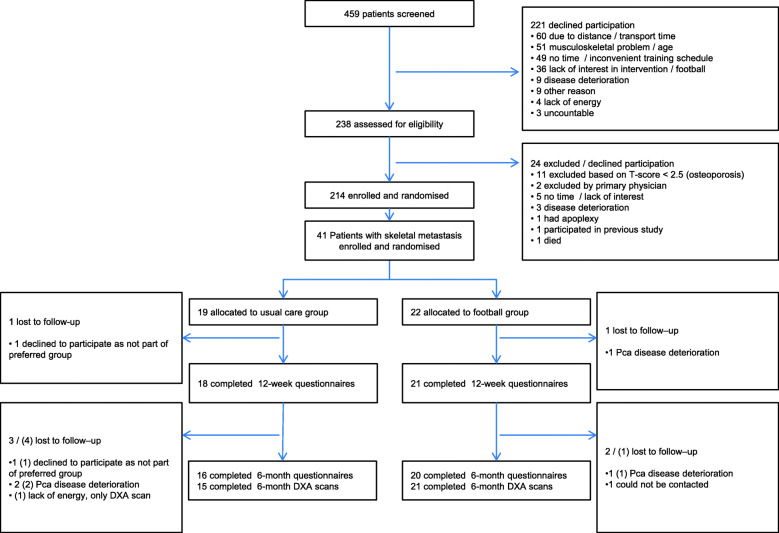
Flow of participants. DXA dual-energy X-ray absorptiometry, Pca prostate cancer, () DXA lost to follow-up

Retention for the 41 patients with skeletal metastatic disease was 95% at 12 weeks (FG = 95%, UC = 95%) and 88% at 6 months (FG = 91%, UC = 84%) (Fig. 1). Median attendance in FG from baseline to 12 weeks was 13 sessions (interquartile ratio 10, 63%). From baseline to 6 months, it was 23 sessions (interquartile ratio 23, 54%). On average, football sessions lasted 58.8 min, see further details [10].

### Outcomes

Table 2 presents the safety outcomes for the adverse events that occurred in the two groups from baseline to 6 months. No significant differences were found between groups, although UC had four hospital admissions and FG had none (*P* = 0.078).

**Table 2:** Safety outcomes

Safety outcomes		Football group (***n*** = 22)	Usual care group (***n*** = 19)
Falls		1	1
*P*-value	1.00		
Fractures		0	0
*P*-value	1.00		
Hospital admissions			
One		0	2
Two		0	1
Total		0	4
*P*-value	0.078		

Table 3 summarises mean changes for primary and secondary outcomes (see Additional file 1). As shown, the primary outcome, PCa-specific QoL, was significantly higher for FG compared to UC at 12 weeks, with a 7.6-point difference (95% CI, 0.5 to 15.0; *P* = 0.038). No statistically significant changes were found in the secondary outcomes. Exploratory analysis found disease progression in 25 out of 41 patients, with significantly more cases of disease progression in UC (16 patients out of 19) compared to FG (9 out of 22; *P* = 0.009).

**Table 3:** Effect outcomes

	Football group (***n*** = 22)		Usual care group (***n*** = 19)		Covariance analysis, difference between groups, mean (95% CI)	
	*n*	Mean change (95% CI)	*n*	Mean change (95% CI)	Adjusted for age and baseline score	*P*-value
Change in prostate cancer-specific quality of life (points, higher is better)						
12 weeks	21	− 1.8 (− 6.8 to 3.3)	18	− 9.0 (− 14.4 to − 3.5)	7.6 (0.5 to 15.0)	0.038
6 months	20	− 5.5 (− 10.6 to − 0.4)	16	− 5.8 (− 11.5 to − 0.1)	0.5 (− 7.3 to 8.4)	0.895
Change in lean body mass (kilogrammes)						
6 months	21	− 0.3 (− 1.1 to 0.5)	15	− 0.4 (− 1.3 to 0.6)	− 0.2 (− 1.4 to 0.9)	0.673
Change in fat mass (kilogrammes)						
6 months	21	− 0.4 (− 1.3 to 0.6)	15	− 0.2 (− 1.4 to 1.0)	0.4 (− 1.1 to 1.8)	0.610
Change in total hip bone mineral density (g/cm^2^)						
6 months	21	0.007 (− 0.025 to 0.039)	15	0.039 (0.001 to 0.078)	− 0.028 (− 0.083 to 0.026)	0.295
Change in lumbar spine L1–L4 bone mineral density (g/cm^2^)						
6 months	21	0.045 (− 0.005 to 0.096)	15	0.017 (− 0.042 to 0.077)	0.029 (− 0.055 to 0.113)	0.487
Change in general physical health (SF-12)						
12 weeks	21	− 2.0 (− 4.9 to 0.9)	18	− 3.7 (− 6.8 to − 0.6)	1.9 (− 2.4 to 6.2)	0.384
6 months	20	− 3.3 (− 7.4 to 0.7)	16	− 3.7 (− 8.3 to 0.8)	0.3 (− 6.0 to 6.7)	0.912
Change in general mental health (SF-12)						
12 weeks	21	0.1 (− 3.1 to 3.2)	18	− 2.0 (− 5.4 to 1.4)	1.9 (− 2.6 to 6.4)	0.402
6 months	20	− 2.4 (− 6.3 to 1.4)	16	− 2.9 (− 7.4 to 1.6)	0.1 (− 5.9 to 6.1)	0.976

*SF-12* 12-Item Short Form Health Survey

## Discussion

Our subgroup analysis on mPCa patients participating in the FC Prostate Community Trial showed that the risk of falls, fractures and hospital admissions was not higher in FC compared to UC. This indicates that physical exercise alone does not pose a threat in men with skeletal metastatic lesions and can improve physical function and QoL. Notably, the magnitude of difference in PCa-specific QoL between the two groups is > 6 points, which is the established minimum clinically important difference in patients with advanced PCa [13]. In line with our results, Cheville et al., who evaluated exercise delivered as telerehabilitation with 264 participants (51%) with bone metastases, found significant positive effects on QoL [14]. Additionally, both studies found reduced hospital utilisation (e.g. reduced length of stay or fewer hospital admissions) when all of the participants were analysed [11, 14].

The benefits of the intervention on QoL reported in the current study were not found in our primary analysis, which included participants without metastatic disease, indicating that football may be a way to increase QoL in men with advanced PCa only. However, it could also indicate that FACT-P may be a more suitable outcome measure and may have higher responsiveness in patients with advanced disease. In comparison, a previous study by Segal et al. showed that participants treated with palliative intent (i.e. patients with advanced/metastatic disease) reported a difference of 5.7 points on FACT-P and also showed larger effects in comparison to men treated with curative intent [15].

The major limitation of this subgroup analysis is that the number of patients is low. Albeit limited by the number of patients and events, the analysis did not indicate that physical exercise increases the risk of injury and hospitalisation in men with skeletal metastases. The reported difference in disease progression favouring FG should be interpreted with caution as the finding can be spurious, and the outcome was exploratory. Moreover, the sample size is small. Lastly, because patients with unstable and/or painful skeletal metastasis likely did not participate in this trial due to self-exclusion or the exclusion criteria, the results cannot be generalised to this population. Further research should focus on the safety aspects of exercise modalities or modifying them for cancer patients with skeletal metastatic disease, the presence of osteoporosis or skeletal pain treated with opioids, which often precludes patients from engaging in exercises. Stability and size of metastases should be explored in future studies to further evaluate the effects of the loading during exercise.

## Conclusions

In this study, the patients with skeletal mPCa enrolled in a physical exercise programme experienced better QoL without increased adverse events, which indicates that high-impact, community-based aerobic exercise in this clinical subgroup is safe. Based on these findings, trials are warranted to confirm the safety of exercise more broadly in cancer patients with skeletal metastatic disease.

## Supplementary Information

**Additional file 1: Supplementary Table 1.** Means of outcomes at baseline, 12 weeks and 6 months

## Data Availability

Please contact the corresponding author for data requests.

## Abbreviations

CI: Confidence interval
FACT-P: Functional Assessment of Cancer Therapy-Prostate
ISUP: International Society of Urological Pathology
mPCa: Metastatic prostate cancer
PCa: Prostate cancer
QoL: Quality of life
RCT: Randomised controlled trial
SF-12: 12-Item Short Form Health Survey
