# Supplementary material for: Safety and Effects of Football in Skeletal Metastatic Prostate Cancer: a Subgroup Analysis of the FC Prostate Community Randomised Controlled Trial
Source: Sports Med Open. 2021 Apr 20;7:27. doi: 10.1186/s40798-021-00318-6 (PMC8058127; doi:10.1186/s40798-021-00318-6)
Supplement: Supplementary file 1 — Additional file 1: Supplementary Table 1. Means of outcomes at baseline, 12 weeks and 6 months [file 40798_2021_318_MOESM1_ESM.pdf]

Electronic Supplementary Material

This supplementary material has been provided by the authors to give readers additional information about their work.

Supplement to: Eik Dybboe Bjerre[1], Sarah Weller[2], Mads Hvid Poulsen[3,], Søren Sørensen Madsen[4], Rie Dybboe Bjerre[5], Peter Busch Østergren[6], Michael Borre[7], Klaus Brasso[8] and Julie Midtgaard[9]. Safety and effects of football in skeletal metastatic prostate cancer: A subgroup analysis from the 'FC Prostate Community' randomized controlled trial. Sports Medicine - Open.

Corresponding author: Eik Dybboe Bjerre; eik.dybboe.bjerre@regionh.dk, telephone: +4535457917 ORCID ID: 0000-0001-9370-5162

AUTHORS AFFILIATIONS AND ADDRESSES

[1] The University Hospitals' Centre for Health Research, Copenhagen University Hospital Rigshospitalet, Department 9701, Blegdamsvej 9, DK-2100 Copenhagen Ø, Denmark, eb@ucsf.dk

[2] Prostate Cancer Supportive Care Program, Vancouver Prostate Centre, Vancouver, Canada, sweller@prostatecentre.com

[3] Department of Urology, Odense University Hospital, Odense, Denmark and Academy of Geriatric Cancer Research, Odense University Hospital, Odense, Denmark, mads.poulsen@rsyd.dk

[4] Department of Urology, Hospital of Southwest, Denmark/Esbjerg, Esbjerg, Denmark, soeren.soerensen.madsen@rsyd.dk

[5] Herlev-Gentofte Hospital, University of Copenhagen, Hellerup, Denmark, rie.dybboe.bjerre@regionh.dk

[6] Department of Urology, Herlev and Gentofte University Hospital, Herlev, Denmark, peter.busch.oestergren@regionh.dk

[7] Department of Urology, Aarhus University Hospital, Aarhus, Denmark, borre@clin.au.dk

[8] Copenhagen Prostate Cancer Center, Department of Urology, Rigshospitalet, University of Copenhagen, Copenhagen, Denmark, klaus.brasso@regionh.dk

[9] The University Hospitals' Centre for Health Research, Rigshospitalet, Copenhagen, Denmark and Department of Public Health, University of Copenhagen, Copenhagen, Denmark, julie.midtgaard.klausen@regionh.dk

Supplementary Table 1. Means of outcomes at baseline, 12 weeks and 6 months

	Baseline			12 weeks			6 months		
	n	Mean	SD	n	Mean	SD	n	Mean	SD
Prostate cancer-specific quality of life (points, higher is better)									
FG	22	124.2	18.9	21	122.5	18.8	20	119.2	20.4
UC	19	121.2	15.5	18	112.7	16.4	16	117.8	17.3
Lean body mass (kilograms)									
FG	22	53.6	7.3		NM		21	53.6	7.3
UC	19	58.5	6.0				15	57.7	5.2
Fat mass (kilograms)									
FG	22	29.2	8.6		NM		21	29.0	10.3
UC	19	33.1	9.1				15	32.2	9.1
Total hip bone mineral density (grams/cm^2)									
FG	22	1.101	0.122		NM		21	1.141	0.202
UC	19	0.981	0.143				15	0.985	0.147
Spine bone mineral density (grams/cm^2)									
FG	22	1.184	0.281		NM		21	1.235	0.356
UC	19	1.317	0.306				15	1.343	0.342
General physical health (SF-12)									
FG	22	48.8	8.9	21	46.5	9.0	20	45.4	10.1
UC	19	50.2	6.9	18	46.7	8.7	16	47.6	10.2
General mental health (SF-12)									
FG	22	53.0	6.1	21	53.1	7.7	20	50.9	11.1
UC	19	51.9	8.0	18	50.1	6.5	16	49.6	8.2

Abbreviations: SD, standard deviation; NM, not measured.
